# Examining the Relationship Between Community Integration and Mental Health Characteristics of Individuals With Childhood Acquired Neurological Disability

**DOI:** 10.3389/fped.2021.767206

**Published:** 2021-11-22

**Authors:** Christine Nguyen, Abigail Leung, Andrea Lauzon, Mark T. Bayley, Laura L. Langer, Dorothy Luong, Sarah E. P. Munce

**Affiliations:** ^1^Department of Occupational Therapy & Occupational Science, University of Toronto, Toronto, ON, Canada; ^2^Toronto Rehabilitation Institute, University Health Network, Toronto, ON, Canada; ^3^Holland Bloorview Kids Rehabilitation Hospital, Toronto, ON, Canada; ^4^Lawrence Bloomberg Faculty of Nursing, University of Toronto, Toronto, ON, Canada; ^5^Faculty of Medicine, University of Toronto, Toronto, ON, Canada; ^6^Rehabilitation Sciences Institute, University of Toronto, Toronto, ON, Canada; ^7^Institute of Health Policy, Management & Evaluation, University of Toronto, Toronto, ON, Canada

**Keywords:** cerebral palsy, acquired brain injury, depression, anxiety, community integration, transitional care services, Patient Health Questionnaire-4, Community Integration Questionnaire

## Abstract

**Background:** Many individuals with cerebral palsy (CP) or acquired brain injury (ABI) are at higher risk of lowered psychosocial functioning, poor mental health outcomes and decreased opportunities for community integration (CI) as they transition to adulthood. It is imperative to understand the characteristics of those at highest risk of dysfunction so that targeted interventions can be developed to reduce the impact.

**Methods:** This quantitative, cross-sectional study examines current patients of the Living Independently Fully Engaged [(LIFEspan) Service], a tertiary outpatient hospital-based clinic. The Patient Health Questionnaire-4 (PHQ-4) and the Community Integration Questionnaire (CIQ) were administered to participants. Personal health information was also collected from participants' health charts, and participant interviews. Associations of sex and condition with the outcomes of screening for further assessment of depression, screening for further assessment of anxiety, and CI were calculated using *t*-tests and Chi-square tests.

**Results:** 285 participants completed standardized screening tools for depression and anxiety (PHQ-4) and 283 completed the Community Integration Questionnaire (CIQ). Mean age was 23.4 (4.2) years; 59% were diagnosed with CP, 41% diagnosed with ABI, and 56% were male. A moderate proportion of the sample screened positive for further assessment of anxiety (28%) and depression (16%), and the overall mean score on the CIQ for the sample was 15.8 (SD 5.1). Participants that screened positive for further assessment of depression and anxiety on the PHQ-4 had lower scores on the Social Integration subscale of the CIQ (*p* = 0.04 and *p* = 0.036, respectively). Females were found to have significantly higher community integration than males (*p* = 0.0011) and those diagnosed with ABI were found to have significantly higher community integration than those with CP (*p* = 0.009), respectively. A weak negative association was found between age for the total sample and overall PHQ-4 score (*p* = 0.0417). Presence of an intellectual or learning disability/challenge was associated with a lower CIQ score (*p* = 0.0026).

**Conclusions:** This current study, highlights the need for further research to explore the unique needs and barriers faced by this population. This study may inform assessments and interventions to support the mental health and community integration of this population.

## Introduction

With advances in medical care, ninety percent of children with childhood-onset disability will now survive into adulthood (1). However, more than half report receiving inadequate support and services during adolescence and the transition to adult healthcare (1). Adolescents with childhood-onset disability transitioning from the pediatric to the adult healthcare system are particularly vulnerable as their medical and mental health services may become scarcer due to the fact that many adult programs target recent, not childhood-onset conditions (2, 3). Other barriers these individuals may face include a lack of specialist clinicians, their own financial limitations, or a lack of institutional support (e.g., age-cut off policies or insurance policies) (3). Without appropriate services, health concerns may remain undetected, putting adolescents at increased risk of developing preventable secondary physical and mental health complications and comorbidities (4). The transition process is further complicated as young adults with disabilities may be at risk for additional psychosocial difficulties associated with the adolescent stage including poor social functioning, anxiety disorders, depression, suicidal ideation, and suicide attempts (5–10).

Among the most common childhood-onset disabilities are cerebral palsy (CP) and acquired brain injury (ABI) (11). CP is the leading cause of physical disability in childhood affecting 2–2.5 per 1,000 live births (2). It includes a group of permanent conditions that affect the development of movement and posture (11). It is caused by non-progressive disturbances that have occurred in the developing fetal or infant brain (11). Impairments may include physical impairments such as hemiparesis, diplegia or quadriplegia, speech disorders, sensory deficits, intellectual disabilities and seizures (11). Childhood-onset ABI is an injury to the brain that is not due to hereditary or degenerative causes, and may have a traumatic or non-traumatic cause that may also negatively impact individual development and function (12).

Many individuals with CP or ABI experience long-term physical, cognitive, emotional, and behavioral problems that are substantial barriers to achieving formal post-secondary education, economic independence, and social inclusion needed for a successful transition to adulthood (12). For individuals with these childhood-onset conditions, their transition into adulthood is not only marked by changes to musculoskeletal disabilities, but also changes in psychological and social development (13). The combined effects of the disability with the physical, emotional, and social changes that accompany typical transitions into adolescence create an added burden to individuals with these conditions. Without addressing these unique needs, individuals may be at higher risk of lowered psychosocial functioning, such as poor mental health outcomes and decreased opportunities for community engagement and participation (14, 15).

Until recently, the mental health characteristics among young adults (i.e., 18 years and older) with childhood onset disabilities, including CP and childhood onset ABI, have not been well-characterized. Thus, the objectives of the current study are to describe the mental health characteristics i.e., anxiety, depression, as well as community integration (CI) of adults with CP and childhood-onset ABI, including: examining whether there are sex differences in the levels of CI and proportions of positive screens for further assessment of depression and anxiety among adults with CP and childhood-onset ABI; examining whether there are condition-specific differences (CP vs. ABI) in the levels of positive screens for further assessment of depression, positive screens for further assessment of anxiety, and CI among adults with CP and ABI; examining whether there is association between CI and scores on measures screening for further assessment of depression and anxiety; and examining other variables such as severity of condition, age, and cognitive ability and their relation to CI and scores on measures screening for further assessment of depression and anxiety. A better understanding of these associations could help to inform the development of targeted mental health interventions and programs for this population.

## Methods

### Study Design

This is a cross-sectional study of individuals that are current patients of a transitional care service called the Living Independently Fully Engaged (LIFEspan) Service. The Strengthening the Reporting of Observational Studies in Epidemiology (STROBE) Statement guidelines for reporting observational studies were followed (16).

### Setting

The LIFEspan Service aims to target the unique psychosocial challenges faced by adolescents with CP and ABI and represents a partnership service between Toronto Rehabilitation Institute at the University Health Network (TRI-UHN) and Holland Bloorview Kids Rehabilitation Hospital in Toronto, Ontario, Canada. The LIFEspan Service is a multi-disciplinary service delivery model for individuals with childhood-onset neurological disabilities, including CP and ABI. The LIFEspan Service aims to bridge the gap for these individuals as they transition from pediatric to adult rehabilitation systems. It aims to deliver continuous coordinated care to support physical, emotional, as well as functional development of this population. In particular, this service offers programs and services that target prevention and management of mental health and psychosocial development. Specific programs include physical activity engagement, social and communication skill building, and self-management skills, with an overarching goal of promoting independent skill building and self-advocacy skills to increase community engagement.

### Participants and Recruitment

Eligible participants included individuals who (1) were >17 years of age; (2) registered with LIFEspan Service; (3) had a diagnosis of CP or childhood-onset ABI; and (4) were able to comprehend and communicate in English or have an interpreter or use augmentative and alternative communication. Individuals who had severe intellectual disability or could not answer the questionnaires were excluded from the study.

Participants were recruited in person by a team member working at the LIFEspan Service who was not part of the participants' immediate circle of care to minimize conflicts of interest. A convenience sample was used, as participants were recruited while they were at TRI-UHN (i.e., for clinic visits). All participants were recruited between September 2017 and August 2019.

### Data Collection and Database Items

Demographic variables included the participants' sex, age, and diagnosis of CP or ABI.

Other variables collected included the participants' use of assistive devices, education level, co-morbid medical conditions, intellectual/learning disabilities or learning challenges, medications, and severity of condition. The Gross Motor Function Classification System (GMFCS) was collected to describe the severity of CP patients, but due to the heterogeneity of the cause of brain injuries, no specific information was collected for severity of ABI patients.

Standardized measures for screening depression and anxiety and CI were used for this study. The assessments were the Patient Health Questionnaire-4 (PHQ-4) and the Community Integration Questionnaire (CIQ). They were completed by the participant while waiting for the clinic appointment and reviewed by the LIFEspan Nurse Practitioner (AL) during the participant's scheduled clinic appointment.

### Data Items and Measures

#### PHQ-4

The PHQ-4 (17) is a brief and valid screening tool for further assessment of depression and anxiety. It has a 4-item inventory (2 for anxiety and 2 for depression) that is rated on a 4 point Likert-type scale. It provides separate scores for anxiety and depression. Each item is scored as 0- not at all, 1- several days, 2- more than half the days, and 3- nearly every day. The PHQ-4 has been correlated with mental health (*r* = 0.80), social functioning (*r* = 0.52), general health perceptions (*r* = 0.48), role functioning (*r* = 0.37), bodily pain (*r* = 0.36), and physical functioning (*r* = 0.36). Anxiety is defined as a score higher than or equal to 3 on the Anxiety subscale of the PHQ-4 (questions 1 and 2), and depression is defined as a score of higher or equal to 3 on the Depression subscale (questions 3 and 4). Scores are rated as normal (0–2), mild (3–5), moderate (6–8), and severe (9–12) and indicate the level of psychological distress.

#### CIQ

The CIQ (18) is a 15-item inventory developed to measure levels of community integration. It has typically been used among adults following diagnosed traumatic brain injuries. Community integration is defined as “integration into a home-like setting, integration into a social network, and integration into productive activities such as employment, school or volunteer work.” The CIQ has demonstrated good test-retest reliability and internal consistency. The assessment score ranges from 0 to 29, where the total score is calculated using a summation of the scores from individual questions. A higher score indicates greater integration, whereas a lower score reflects less integration. The CIQ can be divided into three subscales which specifically measure at Home Integration (items 1–5), Social Integration (items 6–11), and Productivity (summation of item 12 and the Productivity variable). While the CIQ has increasingly been used in studies with non-brain injury populations, including CP, there measure has not actually been validated for use in CP populations.

### Data Analysis

Descriptive statistics of the participants' characteristics, socio-demographic factors, and assessment scores were calculated. Participants' scores on the PHQ-4 and the CIQ were compared by sex and condition (i.e., CP or ABI). *T*-tests, Chi-square, Fisher's exact, or ANOVA tests were used to determine group differences (i.e., sex, condition, severity of condition, and presence of intellectual/learning disability/challenge) in the levels and proportions of anxiety and depression, as well as group differences in overall CIQ score. A Spearman ranked correlation was performed between depression and anxiety composite scores on the PHQ-4 and the CIQ. Statistics were calculated using SPSS version 25. Alpha was set to 0.05.

### Research Ethics

This study was approved by the Research Ethics Board at University Health Network, ID# 16-5875. All patients provided written informed consent to have the data from the PHQ-4 and CIQ entered into the database.

## Results

### Demographic and Clinical Characteristics

There were 285 participants that were recruited for the study over the course of almost 24 months (the LIFEspan Service sees ~250 eligible participants per year). The average age was 23.4 (4.2 SD) years and 56% of the sample was male. The majority of the sample was single (96%) and lived with family members (92%). There was a diverse range of education levels within the sample, including participants who were currently attending or had finished secondary school as well as participants who were currently completing or had finished a postgraduate degree. Additionally, employment status varied within the sample, including participants who had full-time or part-time work, were students, had a volunteer position, or were on disability income.

Fifty nine percent of the sample had a diagnosis of CP. There was a wide range of mobility needs within the population, with all participants reporting that they used some type of assistive devices such as a brace or a power wheelchair. More than half (60%) reported having some form of concurrent intellectual/learning disability or learning challenges. The demographic and clinical characteristics of the sample population are described in Table 1.

**Table 1:** Demographic and clinical characteristics of patients of the LIFEspan service.

**Characteristic**	***N* = 285**
	***n* (%)**
**Age**	
Mean (SD)	23.4 (4.2)
Median	23
Interquartile range	20–26
**Sex**	
Male	160 (56)
Female	125(44)
**Relationship status**	
Married/common-law	11 (4)
Single	274 (96)
**Education**	
Currently in or finished high school	106 (37)
Currently attending college or university	79 (28)
Finished college or university	79 (28)
Currently in/finished postgraduate degree	3 (1)
Unknown	5 (2)
**Employment**	
Working FT	44 (16)
Working PT	48 (17)
Student	104 (37)
Volunteering	17 (6)
Unemployed	11 (4)
On disability	56 (20)
**Use of assistive devices/mobility aids**	
Brace	62 (22)
Cane	23 (8)
Walker	38 (13)
Scooter	7 (2)
Wheelchair	29 (10)
Power wheelchair	57 (20)
**Diagnosis**	
Cerebral palsy (CP)	167 (59)
Acquired brain injury (ABI)	118 (41)
**PHQ-4 screen**	
Anxiety (positive screen)	79 (28)
Depression (positive screen)	47 (16)
**Intellectual disability, learning disability or learning challenges**	
Yes	168 (60)
No	113 (40)
**Falls**	
Yes	76 (27)
No	207 (73)
**Living situation**	
Family/spouse	262 (92)
Alone	10 (4)
University/college residence	9 (3)
With roommates	4 (1)
**Pain**	
Yes	126 (45)
No	157 (55)

### Community Integration and Depression and Anxiety

The overall mean score on the CIQ was 15.8 (SD 5.1). Based on the PHQ-4, 28% of this population screened positive for further assessment of anxiety, and 16% screened positive for further assessment of depression. There was a weak negative Spearman correlation between the CIQ score and depression screening composite score on the PHQ-4 (rho = −0.13, *p* = 0.032). Participants that screened positive for further assessment of depression on the PHQ-4 had lower scores on the Social Integration (*p* = 0.04) and Productivity subscales (*p* = 0.0012) of the CIQ. Social Integration was also lower for participants that screened positive for further assessment of anxiety on the PHQ-4 than those that did not (*p* = 0.036) (see Table 2 for CI differences by mental health status).

**Table 2:** Community integration differences by mental health status.

		**Mean**	**Standard deviation**	***p*-value**
**Depression—positive screen for further assessment**				
CIQ score	Yes	14.6	5.9	0.075
	No	16.0	5.0	
Home integration	Yes	3.3	2.3	0.74
	No	3.4	2.3	
Social integration	Yes	6.7	2.3	0.04^*^
	No	7.4	2.0	
Productivity	Yes	3.9	2.2	0.012^*^
	No	4.7	1.9	
**Anxiety—positive screen for further assessment**				
CIQ score	Yes	15.6	5.4	0.67
	No	15.9	5.1	
Home integration	Yes	3.4	2.4	0.91
	No	3.4	2.2	
Social integration	Yes	6.9	2.2	0.036^*^
	No	7.5	2.0	
Productivity	Yes	4.5	2.1	0.74
	No	4.6	1.9	

* *represents statistical significance*.

### Differences by Sex in Community Integration, Depression, and Anxiety

Female participants were significantly more integrated in their community compared to male participants, as demonstrated by higher total CIQ scores (*p* = 0.0011) and higher scores on the Home (*p* = 0.002) and Social Integration subscales (*p* = 0.015). There were no sex differences in the proportions of positive screens for further assessment of depression or positive screens for further assessment of anxiety. Sex differences in CI scores and proportions of positive screens for further assessment of depression and anxiety have been reported in Tables 3.1, 3.2.

**Table 3.1:** Sex differences in the CIQ and PHQ-4.

	***n***	**Mean**	**Standard**	***p*-value**
			**deviation**	**(2-tailed)**
CIQ total score	283	15.8	5.1	
Male	158	14.9	5.2	0.0011*
Female	125	17.9	4.9	
CIQ home integration	283	3.4	2.3	
Male	158	2.9	2.3	0.0002*
Female	125	3.9	2.2	
CIQ social integration	283	7.3	2.1	
Male	158	7.1	2.2	0.015*
Female	125	7.7	2.0	
Productivity	283	4.6	1.9	
Male	158	4.5	1.9	0.52
Female	125	4.7	2.0	
Total PHQ-4 score	285	2.9	3.0	
Male	160	2.7	3.1	0.14
Female	125	3.2	2.8	

Equal variances assumed. *indicates statistical significance.

**Table 3.2:** Sex differences in screening for further assessment of depression and anxiety.

	**Positive screen**	**Negative screen**	**χ^**2**^**,
	**depression *n* (%)**	**depression *n* (%)**	***p*-value**
Male	136 (86)	24 (14)	
Female	102 (82)	23 (18)	χ^2^(1) = 0.39, *p* = 0.53
	**Positive screen**	**Negative screen**	***χ***^**2**^,
	**anxiety** ***n*** **(%)**	**anxiety** ***n*** **(%)**	***p*** **-value**
Male	123 (77)	37 (23)	
Female	83 (66)	42 (34)	χ^2^(1) = 1.41, *p* = 0.23

### Differences by Condition in Community Integration, Depression, and Anxiety

Participants with ABI reported significantly higher scores (i.e., greater integration) on their total CIQ scores compared to participants with CP (*p* = 0.009), and significantly higher scores on the Social Integration (*p* = 0.007) and Productivity (*p* = 0.02) subscales of the CIQ. No condition-specific differences were found for the total PHQ-4 score or in the proportions of positive and negative screening for further assessment of depression or anxiety. Differences by condition in CI scores and proportions of positive and negative screening for further assessment of depression and anxiety symptoms have been reported in Tables 4.1, 4.2.

**Table 4.1:** Condition-specific differences in the CIQ and PHQ-4.

	***n***	**Mean**	**Standard**	***p*-value**
			**deviation**	**(2-tailed)**
CIQ total score	283	15.9	5.1	
CP	165	15.1	5.2	0.009**
ABI	118	16.7	5.0	
CIQ home integration	283	3.4	2.3	
CP	165	3.2	2.2	0.059
ABI	118	3.7	2.4	
CIQ social integration	283	7.3	2.1	
CP	165	7.0	2.0	0.007**
ABI	118	7.7	2.2	
CIQ productivity	283	4.6	1.9	
CP	165	4.4	2.0	0.02*
ABI	118	4.9	1.7	
Total PHQ-4 score	285	2.9	3.0	
CP	167	2.9	3.2	0.99
ABI	118	2.9	2.7	

* and **indicates statistical significance.

**Table 4.2:** Condition-specific differences in screening for further assessment of depression and anxiety.

	**Positive screen**	**Negative screen**	**χ^**2**^**,
	**depression *n* (%)**	**depression *n* (%)**	***p*-value**
CP	138 (83)	29 (17)	
ABI	100 (85)	18 (15)	χ^2^(1) = 0.088, *p* = 0.77
	**Positive screen**	**Negative screen**	***χ***^**2**^,
	**anxiety** ***n*** **(%)**	**anxiety** ***n*** **(%)**	***p*** **-value**
CP	118 (71)	49 (29)	
ABI	88 (75)	30 (25)	χ^2^(2) = 0.24, *p* = 0.63

### Age and Community Integration, Depression, and Anxiety

No association was found between age and CIQ score. A Spearman ranked correlation test found a weak negative association between age for the total sample and overall PHQ-4 score (ρ = − 012, *p* = 0.0417), however, no associations were found when analyzing by condition.

### GMFCS and Community Integration, Depression, and Anxiety

The highest proportion of CP participants were classified in Level 1 (33%) or Level 4 (34%) of the GMFCS. When collapsing the GMFCS into three categories of mild (Level 1), moderate (Level 2 and Level 3), and severe (Level 4 and Level 5), the highest proportion of participants were found in the severe category (42%). The complete breakdown is reported in Table 5. An ANOVA found no relationship between GMFCS level and CIQ score, and Chi-square tests found no relationship between GMFCS levels and the proportions of positive and negative screening for further assessment of depression or anxiety.

**Table 5:** GMFCS breakdown in CP participants.

**Characteristic**	***N* = 164**
	***n* (%)**
**Levels of GMFCS**	
Level 1	54 (33)
Level 2	19 (12)
Level 3	22 (13)
Level 4	56 (34)
Level 5	13 (8)
**Collapsed levels of GMFCS**	
Mild (Level 1)	54 (33)
Moderate (Level 2 & 3)	41 (25)
Severe (Level 4 & 5)	69 (42)

### Cognitive Challenges and Community Integration, Depression, and Anxiety

Those with an intellectual or learning disability/challenge had a lower mean score on the CIQ (15, SD 5.28) than those without (17, SD 4.76). A *T*-test found the difference in means to be significant (*p* = 0.0026). Chi-square tests found no relationship between presence of intellectual or learning disability/challenge and the proportions of positive and negative screening for further assessment of depression or anxiety.

## Discussion

This present study is one of the first to describe the relationship between mental health (anxiety symptoms, depression symptoms) and CI amongst adults with CP and childhood-onset ABI. An additional strength included examining sex and condition-specific differences in depression, anxiety, and CI among adults with CP and childhood-onset ABI. The results demonstrated that participants that screened positive for further assessment of depression on the PHQ-4 had lower scores on the Social Integration and Productivity subscales of the CIQ. Those that screened positive for further assessment of anxiety on the PHQ-4 also had lower scores on the Social Integration subscale of the CIQ. Females were found to be more integrated in the community when compared to their male counterparts, and individuals with ABI had higher CI scores (i.e., were more integrated) than those with CP. Those with an intellectual or learning disability/challenge had lower CIQ scores. Our findings did not demonstrate any significant differences with respect to sex and presence of depression or anxiety symptoms, condition and presence of depression or anxiety symptoms, severity of condition and presence of depression or anxiety symptoms, or presence of intellectual or learning disability/challenge and presence of depression or anxiety symptoms.

### Levels of Depression and Anxiety in LIFEspan Participants

Our study found that 17% of the sample with CP had a PHQ-4 score consistent with a positive screen for further assessment of depression; this finding is fairly consistent with some existing evidence that indicates that 20–25% (19, 20) of adults with CP have clinically significant levels of depressive symptoms, as assessed by the Child Behavior Checklist (19) and the Epidemiological Studies Depression Scale (20).

A recent study in 501 adults with CP from a US clinic demonstrated that 39% of patients met the criteria for a diagnosis of anxiety disorder (21). This proportion is higher than the 28% of patients in our study with a PHQ-4 score who screened positive for further assessment of anxiety; however, similar to our study, a greater proportion of the study's sample also demonstrated a higher proportion of individuals with CP with anxiety vs. depression symptoms. Furthermore, results from a retrospective longitudinal cohort study demonstrated that individuals with CP had an increased risk of being diagnosed as having depression or anxiety, compared with a matched control group of adults without CP (22). The authors indicated that these results could have been observed because adults with CP experience many physiological, psychological, social, and health-related risk factors that have been shown to be associated with depression and anxiety in the general population such as multimorbidity, increased pain, functional limitations, non-communicable diseases, difficulties with social relationships, and worse sleep (22). Furthermore, and as described previously, poor transitions between pediatric and adult healthcare systems may leave young adults with childhood-onset conditions even more vulnerable to poorer health outcomes and at risk for both physical and mental health complications (3, 13). Lastly, and not surprisingly, the demonstrated proportions of young adults with symptoms consistent with a positive screen for further assessment of depression (16%) and anxiety (28%) in our overall sample were much higher than those reported in the general Canadian population over the age of 15, where 4.7 and 2.5% have self-reported symptoms related to a major depressive episode and generalized anxiety disorder, respectively (23, 24). In fact, it is argued that the proportions demonstrated in our study may have potentially been higher without the programs and supports associated with the LIFEspan Service, which target prevention and management of mental health and psychosocial development. Furthermore, our finding that there was weak negative association between age and PHQ-4 score may indicate the possibility that younger patients with ABI and CP experience more anxiety and depression during the initial transition process, which tapers off with time spent in the LIFEspan service.

The findings that sex was not associated with depression or anxiety among adults with CP and childhood-onset ABI is in contrast to previous research findings in the general population where sex has been shown to play a role in the prevalence of depression and anxiety. For depression, females after puberty experience major depression at roughly twice the rate of males (25). Similarly, for anxiety, the lifetime prevalence of generalized anxiety disorder is found to be 6.6% in women, compared to 3.6% in men (8, 25). This sex difference is found to emerge in mid-adolescence and is associated with hormonal changes during this time period (23). Studies conducted with children with spastic bilateral CP and autism have noted that there were no differences in the prevalence of depression and anxiety found between males and females (20, 26). Further investigations are warranted to confirm whether sex differences exist. Identifying/confirming these differences could inform the development of future interventions to better target and effectively prevent and manage mental health challenges in these populations.

While we did not find any association between condition, or severity of condition and presence or levels of depression and anxiety distress, previous research on depression in individuals with CP has shown that depression or depressive symptoms have been shown to be related to the severity of the condition (2). It may be that the services that LIFEspan patients receive mitigate this relationship; nonetheless this relationship warrants examination in future research.

### Levels of Community Integration in LIFEspan Participants

The current study demonstrated sex differences with respect to levels of CI, with females demonstrating higher levels of integration than males. Previous literature has demonstrated that there are sex differences in the symptoms experienced by adults post-brain injury (27). For example, sex differences in executive functioning post-brain injury have been found, with females performing better than males (28). Higher executive dysfunction in males post-ABI may interfere with their ability to self-regulate and adapt to changes in their environment which may also contribute to their lower scores on the CIQ. Similar sex differences in CI have been previously found in CP. For children with CP, previous literature has found that adolescent girls with CP experience higher levels of participation and enjoyment in social, informal activities compared to adolescent boys with CP (29). Thus, the need to tailor interventions and treatments based on sex differences and experiences should be a consideration for clinicians when working with individuals with childhood-onset conditions.

Cognitive levels may be another important factor to consider for improving community integration. Participants in our study with intellectual or learning challenges/disabilities demonstrated lower CI that those without. This is consistent with research in other populations, such as multiple sclerosis, where higher levels of cognitive impairment were associated with lower scores on the CIQ (30). Interventions will need to be designed with this mind in order to be optimally effective.

Our study also found that individuals with ABI were more integrated within their community compared to individuals with CP, and previous literature is largely consistent with these findings. For example, adolescents and young adults with childhood-onset physical disabilities have reported many barriers to participating in their community (31). Some of these barriers have included lacking feelings of fulfillment and enjoyment, experiencing fatigue and lack of energy, and having complications and fears associated with the condition (31). Individuals with CP, specifically, have reported being poorly integrated in paid employment and sport activities (32). Relatedly, the leisure activities that adults with CP participate in tend to be non-intensive, more passive, home-based, and lack variety (33, 34). Lastly, and as previously noted, social factors including difficulties with social relationships have been shown to be associated with depression and anxiety in the general population; thus, the observed associations between depression and anxiety symptoms and social integration in the current study are not surprising.

### Limitations

We acknowledge some limitations. The generalizability of these results to other youth with CP and ABI are limited due to the selection bias of the sample. All participants of this study were patients in the LIFEspan Service, which offers clinical support and interventions to holistically target the myriad of challenges present in this population. As a result of the resources these individuals have access to, the findings may not be representative of youth with CP and ABI in the general population. Instead, the current sample likely represents a healthier and more engaged group of individuals with CP and ABI.

Another limitation is that this current study did not address the severity of condition of all participants. Lack of information on this variable limits our ability to understand its role and influence on functional outcomes, such as mental health and community integration.

Furthermore, components of the standardized measures chosen may not have been applicable or relevant to our sample population. Most notably, while the CIQ was developed for use in the brain injury population, the psychometric properties for the CP population are not known. Although anecdotally and intuitively, the CIQ items make sense as including important aspects of participation in non-brain injury populations, further validation is needed. Also importantly regarding the CIQ, participants provided feedback indicating that some questions in the CIQ were difficult to interpret. For example, the question, “Do you have a best friend with whom you confide?” was difficult to answer for some participants if they had a group of close friends. This finding alone may represent some of the inherent limitations of psychosocial measures currently used in these populations. In addition, the PHQ-4 was intended as a brief screening tool for depression and anxiety and may not be the most appropriate for this study as it is not a standardized diagnostic tool (18).

### Future Directions

Future research should focus on conducting a longitudinal study starting with a younger population of individuals with CP and childhood-onset ABI using the same objectives. Through tracking and monitoring changes over time, we could identify what can best support these populations as they develop, as well as monitor the effectiveness of the LIFEspan Service. This would provide further insight into psychosocial functioning at different stages of development, as well as the transition of care between pediatric and adult healthcare systems.

Future research should also examine how comorbidities commonly experienced by individuals with CP and ABI impact mental health outcomes. This would be a particularly worthy investigation, as some comorbidities commonly experienced by individuals with ABI and/or CP have been found to have significant impacts on mental health outcomes. For example, it has previously been reported that 20–30% of patients with epilepsy experience symptoms of depression (35); individuals with CP have been found to have a higher frequency of epilepsy compared to matched controls (22). Thus, individuals with co-occurring CP and epilepsy might be at higher risk for depression. A better understanding of factors influencing mental health outcomes will help better develop and target interventions for these populations.

As identified in our study limitations, more research is needed to further explore the impact of severity of conditions on mental health characteristics. Previous literature has suggested that higher severity levels may be associated with more negative function and quality of life for individuals with chronic childhood-onset conditions (2). It would be worthwhile to examine this relationship to identify how to better target and support each specific condition.

Lastly, it would be beneficial to expand the study to include a comparison group of youth with CP and ABI that may not have had access to similar coordinated care services. This would help to elucidate the effects of transitional services such as the LIFEspan Service.

### Conclusions

Our current study, along with previous literature, highlights the need for further research to explore the unique needs and barriers faced by these populations. In addition, our findings may help to inform future assessments, treatment, and interventions to support the mental health and community engagement of individuals with CP and ABI.

## Data Availability Statement

The original contributions presented in the study are included in the article/supplementary material, further inquiries can be directed to the corresponding author/s.

## Ethics Statement

The studies involving human participants were reviewed and approved by University Health Network Research Ethics Board. The patients/participants provided their written informed consent to participate in this study.

## Author Contributions

CN and ALe: led the creation of this manuscript. SM: research project and manuscript creation. All authors contributed to the review and feedback of the manuscript.

## Funding

Funding for this project was provided by an award from the Nurse Practitioners Association of Ontario for Innovations in Chronic Disease Management.

## Conflict of Interest

The authors declare that the research was conducted in the absence of any commercial or financial relationships that could be construed as a potential conflict of interest.

## Publisher's Note

All claims expressed in this article are solely those of the authors and do not necessarily represent those of their affiliated organizations, or those of the publisher, the editors and the reviewers. Any product that may be evaluated in this article, or claim that may be made by its manufacturer, is not guaranteed or endorsed by the publisher.
